# Improving the mental health care process in response to Covid-19 pandemic: The case of a penitentiary mental health division

**DOI:** 10.1371/journal.pone.0293492

**Published:** 2023-10-30

**Authors:** Angela Nuzzi, Valeria Latorre, Domenico Semisa, Barbara Scozzi

**Affiliations:** 1 Department of Mechanics, Mathematics, and Management, Polytechnic University of Bari, Bari, Italy; 2 Complex Organization Unit Psychiatric Diagnosis and Care Service UO San Paolo, ASL Bari, Bari, Italy; 3 Penitentiary Mental Health Service, Department of Mental Health, ASL Bari, Bari, Italy; University of Foggia: Universita degli Studi di Foggia, ITALY

## Abstract

Covid-19 outbreak led all organizations to reorganize their processes to prevent infection and contagion risk. All healthcare facilities, included penitentiary mental health services, had to redesign their processes to safely deliver care services. In this paper, the case of a Penitentiary Mental Health Division located in southern Italy is presented. Soft System Methodology and Business process management principles and techniques are adopted to analyse and redesign the detainees’ mental health care process. The process, characterized by direct, close and prolonged contact with patients, exposes detainees and healthcare staff to a high Covid-19 infection risk. Through document analysis, interviews with the actors involved in the process and direct observation, the process’s inefficiencies and criticalities are identified. The process is redesigned to make it compliant with Covid-19 prevention provisions and national penitentiary regulations and address the other criticalities. The proposed methodological approach–which innovatively combines Soft System Methodology and Business Process Management–constitutes a human-centered process-based redesign approach that can be used both in healthcare and other organizational settings.

## Introduction

The rapid evolvement of coronavirus disease (Covid-19) in a worldwide pandemic situation determined the need to reorganize healthcare systems so as to respond to the increasing demand for assistance in intensive care units and to prevent infection’s diffusion in healthcare facilities [1–3]. Significant endeavours were carried out by healthcare facilities to safely deliver traditional care services and, at the same time, optimal care to patients affected by Covid-19. Several studies discussed the reorganization of the workflows in healthcare facilities in response to the Covid-19 outbreak [4–7].

The present study focuses on the penitentiary mental healthcare system, a highly complex and often neglected domain. Around 970 million people worldwide suffer from some form of mental disorder [8]. World Health Organization (WHO) includes mental health and psychiatry services–that in Italy currently treats about 900,000 adults and 500,000 children–among the essential services that have to be guaranteed in the Covid-19 pandemic scenario [9]. Mental health disorders directly affect Covid-19 people’s susceptibility; people with severe mental health disorders are more likely to suffer from comorbid medical conditions associated with a higher risk of severe Covid-19 illness [10–12]; also, patients with prior psychiatric diagnosis hospitalized for Covid-19 have a higher mortality rate compared with those without a psychiatric diagnosis [13, 14]. Psychiatry services, especially those provided in the penitentiary sector, are fundamental to identify and evaluate mental health disorders that, as just mentioned, represent vulnerability factors for Covid-19 severe illness and infection risk. Therefore, mental health and psychiatry services need to be redesigned to align them to the directives for the prevention of Covid-19 infection, also taking into the account the peculiarities of these services. Psychiatric interventions, which envisage the use of speech (i.e., the main droplets’ producer and vector of infection diffusion [15–17]) as therapeutic and diagnostic element hardly get along with social distancing measures.

Several studies have discussed effective Covid-19 transmission mitigation strategies in correctional facilities, as contact tracing [18], single celling and asymptomatic testing [19], restricting access to essential staff and banning visitors [20], video contact with lawyers and people outside the organization [21], release from prison and home confinement [22]. Although all these measures modified processes and single activities in correctional facilities, to the best of the authors’ knowledge, no study has addressed the issue of redesigning the penitentiary mental health care process in response to the Covid-19 pandemic, but for [23] that, based on a survey conducted in the French penitentiary psychiatric wards, described the organizational measures (e.g., limitation of medical consultations to serious and urgent cases, creation of “Covid units”, cancellation of voluntary psychiatric hospitalizations, reinforcement of preventive hygiene measures and reshuffling of medical staff) implemented to ensure the continuity of care and prevent infection’s spread.

In the paper, the care process of detainees affected by mental disorders is analysed and redesigned by adopting Soft System Methodology (SSM) one of the best-known soft operational research methods [24–27], and Business Process Management (BPM), a discipline which includes principles, methods, modelling techniques and technologies to support the design, enactment, execution, improvement, and monitoring of business processes [28, 29]. Even before the pandemics, BPM was considered a key enabler for the improvement of healthcare processes [30, 31]; healthcare is indeed widely acknowledged as one of the most promising, yet challenging, domains for the adoption of BPM to (re)design processes–both organizational/administrative and clinical ones [32]–so as to provide services with limited resources (in terms of human resources, time and budget) without compromising patient care quality. To achieve the paper goal, actors involved in the process were identified and actively involved, process’s activities were characterized, and a key performance indicator related to Covid-19 infection risk defined. The as-is process was modelled in BPMN, a standard notation that allows the visualization of process’ activities, actors, document and information flows. Based on the analysis of as-is process model, different suggestions were provided and discussed with the actors of the process to identify those changes and improvements considered as feasible and desirable by the actors. Based on that, a to-be process model was developed, tested, and finally implemented. The methodological approach proposed in this paper, by combining SSM and BPM, allows a human-centered process-based redesign approach to be adopted while overcoming the limits of the two approaches.

The paper is organized as follows. An overview of the literature on the adoption of SSM and BPM in healthcare domain is reported in the Background section. In the Material and Methods section, the theoretical lens and the methodological approach adopted are discussed. Then, the case study conducted in the penitentiary mental health division of a Penitentiary Institute located in Bari (Italy) is presented and discussed. Finally, same conclusions are drawn.

## Background

In this section an overview of the literature on (i) healthcare process redesign during pandemics and (ii) the adoption of BPM and SSM to redesign healthcare processes is proposed.

### Healthcare process redesign in response to Covid-19

During Covid-19 pandemic, healthcare facilities faced the challenge of reorganizing healthcare processes to adapt them to the requirements and prescriptions for preventing pandemic infection risk as well as to respond to the increasing demand for assistance from Covid-19 patients. Capalbo et al. [5] developed an Integrated Care Pathway, a well-defined clinical workflow for Covid-19 patients to improve the standard of care and minimize infection diffusion. Strategies and practices for preparedness [3], wards’ reshaping to increase the units’ capacity [33], and specific algorithms to manage patient flow during the pandemic [34] were proposed to redesign Intensive Care Units workflows (ICU). Hospital surgical activities [35–37] were also reorganized to guarantee patients and medical staff safety; for instance, Pelizzo et al. [38] discussed the reorganization of pediatric surgical department workflows to contain infections and maintain continuity of care for children affected by severe diseases: telecommunication and telemedicine were suggested as support modalities for pre- and post-operative care. Essential lifesaving surgical procedures, such as transplantation, were also redesigned to ensure the safety of transplant recipients and health care workers [39, 40]. Emergency departments (ED) were redesigned by introducing infection risk mitigation systems (e.g., ED pre-triage, use of appropriate personal protective equipment, paths differentiation) to provide safe services to Covid-19 patients without compromising other emergency cares [41–43]. Several studies dealt with clinical workflow redesign in other specific specialistic wards; for example, radiotherapy department workflow [44–46], orthopaedic patient workflow [47], oncology practices [48], otolaryngology workflow [49] were revised to respond to Covid-19 pandemic. All contributions generally conduct a descriptive and qualitative analysis of clinical practices and workflows and redesign them based on existing evidence and emerging needs.

Additionally, mental health and psychiatry services were redesigned in response to Covid-19. The rearrangement of psychiatric services was essentially based on the introduction of digital technologies that permit the continuity of psychiatric service delivery with a drastic reduction of Covid-19 infection risk both for patients affected by mental health disorders and medical staff. For instance, Sarcevic et al., [50] provided guidelines to transform inpatient psychiatry service during Covid-19 emergency and suggested investments in e-health technologies to minimize exposure and provide uninterrupted care. Other studies discussed experiences of implementation of digital technologies to adequate and rearrange psychiatric and mental health services during the pandemic. For example, the telepsychiatry service was implemented in psychiatric practice in response to Covid-19 [51], telehealth service was adopted to convert group-level intensive outpatient psychiatric interventions, an important care for high-risk individuals who needed structured psychotherapy group support [52]; a m-Health platform was also developed to support clinical pathways in a child and adolescent neuropsychiatry unit [53].

### BPM and healthcare processes

Business process management includes principles, methods, and mapping techniques to support the analysis and redesign of business processes [28]. To identify previous studies dealing with the application of BPM in the healthcare sector, the research terms (“business process management” OR “BPMN”) AND (“healthcare” OR “healthcare process” OR “clinical process”) were searched in the Title/Keyword/Abstract of documents included in the Scopus database; the search was limited to English studies. On November 30, 2022, 335 results were retrieved. A screening was carried out to exclude those contributions that did not address the objective of the review. A total number of 81 documents were then analysed. Most of them–more than 60%–are conference papers and more than 70% of documents are published in the last seven years year, since 2016.

The review reveals that the adoption of BPM to analyse and redesign healthcare processes is relatively recent, so literature contributions are still limited. BPM was applied to improve healthcare organizational and administrative processes, which are well-structured processes (e.g., patients’ registration, patients discharge, management of laboratory tests, booking processes) necessary to support daily activities in healthcare facilities [54]. For instance, Amantea et al. [55] adopted the BPM approach to analyse the acceptance process of blood bank department in an Italian hospital and improve the process efficiency and effectiveness; BPM was also adopted to analyse the decontamination process in a sterile reprocessing department [56]; to improve the process of delivering genetic test to clinicians in a medical center [57]; to analyse and improve the selection and admission process to the hospital-at-home service [58].

More recently, some scholars have adopted BPM to manage and optimize also clinical processes [31], which are knowledge-intensive and highly dynamic processes related with medical care whose workflow may vary considerably patient by patient (due to, e.g., a sudden change in patients’ conditions). Among them, clinical care pathways in emergency department [59–62]; home healthcare processes [62–65]; clinical process of pediatric kidney transplantation [66]; care process of people affected by multiple sclerosis [67]; care pathways related to chronic obstructive pulmonary disease [68, 69]; breast cancer screening process [70]; care process of people affected by Crohn’s disease [71]; the patient care delivery in multidisciplinary outpatient clinic [72]. In all cases, processes are analysed and redesigned with the goal of improving the process performance. After the analysis of the as-is process, problems and weaknesses are pointed out, avenues for improvement are identified and used as starting points for developing the to-be process. In many cases the to-be process relies on the integration of information systems and technologies. For instance, the care process of chronic patients was analysed and improved by introducing technologies to support patients and clinicians’ activities [73]; the clinical processes of patients affected by amyotrophic lateral sclerosis were redesigned by introducing e-health technologies to monitor patients conditions [74]; digital technologies (e.g. mobile devices) were introduced to improve chest pain clinical guidelines and support clinicians during the enactment of clinical guidelines in the hospital ward [75]; the clinical process of patients affected by hypertension was redesigned by integrating e-health technologies to monitor patients’ conditions [76].

### SSM and healthcare processes

Soft System Methodology (SSM) helps key stakeholders understand the problems they face, and the views held by other stakeholders. It can be used to facilitate negotiation on the actions needed to address problems. SSM is particularly suited for tackling real world problems that are difficult to define and where stakeholders may have divergent views on the situation and the objectives to achieve. SSM has been widely used in other sectors, but not extensively used in healthcare [77]. To identify previous studies that adopt Soft System Methodology in the healthcare domain, a literature review was carried out. The search terms “Soft System Methodology” AND “health*” were searched in the Title/Keyword/Abstract of documents included in the Scopus database, by limiting research to English documents. A total number of 158 documents was retrieved and then screened to eliminate those studies that did not fit the objective of the review. A total number of 69 documents were selected and then analysed. Most selected contributions (around 86%) are case study, namely they present the adoption of SSM to explore problem situation in healthcare domain. Also, more than 70% of the studies are journal article and most of them (more than 60%) are published in the last ten years.

A scoping review on the use and outcomes of SSM in healthcare conducted by Augustsson et al [78] revealed that SSM had most often been used to understand and structure a problem situation and to suggest potential improvements to the situation, but to a lesser extent to implement and evaluate these improvements. For instance, SSM has been adopted to explore patients’ and clinicians’ experience of a specialist epilepsy service delivery and improve it [79] and to explore radiology staff experience to address the problem situation of heavy workloads [80]; Goto [81] used SSM to summarize the current situation of healthcare and long-term care delivery systems, clarify issues and challenges associated with linking these two systems, and propose solutions. Kettlewell et al. [82] applied SSM to study rehabilitation pathways and identify service gap and critical issues. SSM was also adopted to develop an integrated care pathway specifically designed to meet the need of frail and disabled older adults in home care [83]; to support the improvement of patient flows in an outpatient chemotherapy unit [84]; to analyse and identify issues of rehabilitation service for long-term neurological conditions [85]; to improve the patient discharge process [86]; to examine issues in an emergency department and provide potential solutions [87]; to detect organizational problems in a private hospital and try to solve them [88]; to study workflow and information flow in chronic disease care and provide recommendations for design health information technologies to support chronic diseases care [89].

The literature review revealed that SSM was not used in the penitentiary context and no previous contributions combined SSM and BPM approach to analyse and redesign healthcare processes.

## Materials and methods

Process theory is the theorical lens adopted in the paper. Process theory explains how a given outcome develops through a sequence of events and activities [90, 91]. Such a sequence is also defined as a process. More specifically, Harrington [92] defines a process as the set of interdependent activities that take an input, adds value to it, and provides an output to an internal or external customer. The representation of reality as a process embodies a description of how specific outcomes are achieved. Such a description is particularly useful to understand how organizations work and how to improve them [93–96].

The methodological approach more consistent with what prescribed by process theory is Business Process Management (BPM) which may support process theory adoption as it helps identify and graphically represent the reality as chains of activities and events [28, 29]. The BPM discipline envisages some steps that are cyclically executed and that are effectively summarized in the BPM lifecycle [29]:

1. *Process identification and discovery*. In this phase the business problem is posed and the business processes relevant to the problem are identified. The current state (as-is) of the process is analytically studied; to that end, data and information on the process are collected through documentation analysis, interviews with the actors of the process and direct observation. The as-is process is then documented, typically through appropriate process modelling techniques. As to the process modelling techniques, the Business Process Model and Notation (BPMN) which allows the hierarchical structuring of the process and its description at different levels of decomposition [97] is widely used and considered a de-facto standard.
2. *Process analysis*. Critical issues and inefficiencies associated to the as-is process are detected; output of this phase is a collection of criticalities to be addressed.
3. *Process redesign*. In this phase, different redesign strategies aimed to solve and overcome the process’s inefficiencies are proposed, discussed and finally implemented; they may include the definition of a new alternative process workflow, the introduction of new roles and responsibilities and the reassignment of process’ activities to other actors of the process, the introduction of new coordination mechanisms allowing to manage the interdependence between process’ activities [98], and the introduction of digital technologies to innovatively and more effectively manage the process. The redesign strategies may be combined, and multiple to-be alternatives proposed; these alternatives are then simulated on some process pilot instances and the most promising one, i.e. that better meets the performance objectives, becomes the to-be process.
4. *Process implementation*. The to-be process is thus configured (this implies organizational changes as well as the deployment of IT systems or the reconfiguration of the existing ones) and executed.
5. *Process monitoring and control*. Once the redesigned process is running, relevant data are collected and analysed to determine how well the process is performing. Bottlenecks, non-compliance, recurrent errors or deviations with respect to the intended behaviour are identified and corrective actions are undertaken. New issues may arise, requiring the cycle to be repeated on a continuous basis.

BPM may be used to identify the processes carried out and properly manage all the activities that contribute to the creation of a specific output (i.e., a product or service) while paying attention to the needs of the customer, i.e., the person or organizational unit to whom/which the specific output is delivered. Additionally, BPM methods and techniques allow an analytic and incremental approach to process redesign: analytic because changes introduced in the to-be process are the results of an in-depth analysis of the as-is process; incremental because the changes introduced in the to-be process do not radically modify the way process’ actors operate, rather modify and improve only those parts of the process that emerged as critical in the process analysis.

To facilitate the identification of the worldviews and motivations of the actors involved in the process, the Soft System Methodology (SSM) was also considered. Regardless of the application area, Soft System Methodology (SSM) is the most widely used problem structuring methods, consisting in a set of participatory modeling approaches for dealing with unstructured complex problems, which are characterized by the existence of multiple actors, with differing perspectives and conflicting interests [99]. Applications of SSM may be found in multiple different settings [100–106] as well as in the healthcare domain, although SSM has not yet been broadly implemented in the domain analyzed in this study. SSM draws a clear distinction between the real world, wherein a problem situation occurs, and the conceptual world of system thinking, where the problem situation is studied and modelled. To analyse and address a problem, seven steps are proposed [107]:

*Steps 1&2*. *Enter unstructured problem situation & express the problem situation*. The first two steps (real world) deal with the characterization of the problem to be addressed. They require the development of a detailed description of the problem situation that affects the process to be redesigned that can be expressed by a “rich picture”. The rich picture depicts the situation (including the organizational entities of interest, relationships among them, roles and issues of apparent significance) and possible areas of conflict [108].

*Step 3*. *Formulate root definitions of relevant human activity systems*. This step requires succinct descriptions of the system to be studied. The descriptions can be multiple, because the different actors involved in the problem situation may interpret it differently. Each description, defined as root definition, includes the following elements (CATWOE):

- Customer, i.e., the person or organization to whom/which the output is delivered;
- Actors, i.e., the persons that carry out one (or more) activities to deliver the output;
- Transformation, i.e., description of inputs and outputs (e.g., a physical entity, a service, or information);
- Worldview, i.e., reasons why the transformation makes sense;
- Owner, i.e., the person/organization that has the power to terminate the transformation;
- Environment, i.e., the elements that characterize the transformation but are not under the owner’s control.

*Step 4*. *Build conceptual models from the root definitions*. This step requires an in-depth analysis of the root definitions identified. For this purpose, SSM does not prescribe a specific method.

*Step 5*. *Compare models with real world*. This step requires the comparison between the conceptual models developed in step 4 and the real world wherein the problem situation occurs.

*Step 6*. *Define desirable and feasible changes*. This step requires the identification of feasible desirable changes/solutions, which are supposed to be facilitated by the iterative implementation of the previous steps.

*Step 7*. *Take action in problem situations*. The last step requires the implementation of the identified changes/solutions.

The seven steps should be iteratively implemented. Each iteration represents a learning cycle.

The two methodologies–SSM and BPM–were innovatively combined and applied in a case study aimed to analyse and improve the detainees’ mental health care process in response to Covid-19 pandemic. First, actors involved in the management of detainees’ mental health care process in Bari Penitentiary Institute were identified. Then, by adopting techniques envisaged by BPM (i.e., interviews, document analysis and direct observation) and SSM (group interviews and discussions) data collection was carried out. All the information collected was used to define and describe the problem situation to be addressed. A Work Breakdown Structure was developed to graphically show the different process activities. Following the steps of SSM, the problem situation was identified, and the Root Definition (CATWOE) derived to define process’ boundaries and motivations of the analysis. An in-depth analysis of the as-is process model was conducted which included the characterization of process’ activities (input, output, actors and resources were identified), the identification of interdependencies among activities, roles and responsibility, documents and information transferred in the process. The as-is process was modelled in BPMN; the model was validated and discussed with actors involved in the process to detect process inefficiencies and criticalities. Finally, the process was redesigned, and a more effective and efficient process alternative (to-be process) was identified, tested, and implemented.

This study did not specifically involve data collection on human participants as individuals. Data collection (interviews, observations, discussions with actors), carried out by two of the authors directly involved in the process (one of them was the process owner), was rather aimed to the analysis of ordinary activities and procedures. For those reasons, the study did not require any revision and approval by an institutional review board or ethics committee.

### Case study: The mental health care process of detainees in Bari Penitentiary Institute

In this section the case study conducted in the penitentiary mental health division of Bari Penitentiary Institute (Italy) is presented. The detainees’ mental health care process is a core process for the penitentiary mental health division because it addresses psychiatric healthcare needs of the penitentiary population. An instance of the process is started every time a new detainee arrives in the prison or when a suspected psychiatric healthcare need is detected by a penitentiary police officer. After an evaluation of psychiatric conditions conducted by the doctor of Penitentiary Health Area and the identification of the needed psychiatric consultation, the process proceeds with psychiatric visit performed by the psychiatrist who takes in care the detainee if the psychiatric healthcare need is confirmed. Therefore, to be carried out, the process requires multiple competencies belonging to different penitentiary departments, namely Penitentiary Administration, Penitentiary Health Area and Penitentiary Mental Health division, which must work in a coordinated mode.

### Penitentiary mental health division

Mental health care service in Penitentiary Institute of Bari is performed by an organizational division belonging to the Mental Health Department of Bari Local Health Authority.

To describe the division that operates in Bari Penitentiary Institute the model proposed by Daft [109] was adopted. This model envisages contextual dimensions (i.e., goal, environment, dimension, technology, and culture) and structural dimensions (i.e., formalization, specialization, personnel indicators, hierarchy, professionality, centralization) to describe the context in which the organization operates and the structure of the organization, respectively.

#### Goal

The aim of penitentiary mental health division is addressing the healthcare needs of detainees and guaranteeing continuity of care to people that arrive in the penitentiary institute. Within 24 hours by their arrival, the mental health division provides psychological consultation to evaluate mental state, psychological problems and suicide risk of all new prisoners that enter in the penitentiary (this service is called “new entrant service”); it also provides ordinary psychiatric care that may be requested by penitentiary health area at any time during the detention.

#### Environment

Mental health division delivers specialist mental health services in Bari penitentiary institute. As mentioned, the division is part of Mental Health Department of Bari Local Health Authority and collaborates with the Penitentiary Health Area. The environment in which it operates is wide and quite complex because processes unfolded in the mental health division are interdependent with those unfolded by Penitentiary Administration (responsible of the prisoner detention and safety) and the Penitentiary Health Area (responsible of the prisoner’s health).

#### Dimension

Penitentiary mental health division provides services to approximately 450 detainees. In 2019, for example, the penitentiary mental health division was in charge of a total number of 381 detainees, 312 of whom were new entrant detainees. The staff of penitentiary mental health division involves five operators and a coordinator.

#### Technology

Technologies, meant as software and hardware systems to support the division work, are generally limited to administrative processes (for instance, the administrative activities of the mental health division are carried out by using email and web technologies); due to safety requirements, technological support is severely limited during the prisoners assistance; at least in Italy, the adoption of technological devices must be authorized by magistrate and Ministry of Justice (as occurred in the case of the adoption of personal computers with a web connection to allow prisoners to have remote meetings with family during Covid-19 emergency). Soft technologies include all the procedures adopted by psychiatrists to treat the patients. They involve the recognition of the need for treatment, the definition of the diagnostic framework, and the formulation of a pharmacological and psychological therapy.

#### Culture

In 2008, with a Decree of the President of the Council of Ministers (April 1st, 2008), the healthcare expertise was transferred from penitentiary administration to National Health Service. However, penitentiary culture (e.g., organizational practices and conventions) still influences current processes; for example, still now, staff of penitentiary administration often directly requires psychiatry service, as envisaged before 2008.

#### Formalization

Penitentiary mental health division is characterized by a low level of formalization; a limited number of written procedures formally describe the processes of the division (e.g., the memorandum of understanding between Bari Local Health Authority and Bari Penitentiary Institute that regulates the management of prisoner’s self-harm risk).

#### Specialization, professionality, and personnel indicators

Penitentiary mental health division has a high level of specialization: the multi-professional team that operates in the division includes three psychiatrists, a psychologist, a specialist psychologist, and a psychiatric rehabilitation technician; each member of the team plays a specific role based on the professional expertise; a central role is assumed by the coordinator that supervises all the activities of the multi-professional team.

#### Hierarchy

The penitentiary mental health division is part of a vertical hierarchical structure; the division hierarchically depends on the Mental Health Department of Bari Local Health Authority.

#### Centralization

The penitentiary mental health division is characterized by a medium degree of centralization: some decisions are in charge of the coordinator of the division (e.g., decisions involving organizational changes and the management of relationships with magistrate and institutions). Other decisions, especially those related to the assistance of detainees, are made by the members of the healthcare team.

### Detainees’ mental health care as-is process

To understand the problem situation, actors involved in the process were first identified (i.e., Director of the Prison, Deputy Commander of the Penitentiary Police, Director of the Penitentiary Health Area, Doctor of Health Area, psychologists, and psychiatrists). All participants were able to opt out of the study and those who did not participate received the same treatment offered to participants. (Single and group) interviews and discussions with those actors were conducted to develop the rich picture, formulate the root definition and better understand how the as-is process works (step 1–4 of the SSM). An analysis of documentation was also carried out with the aim to define the organizational context, process constraints and existing organizational and operative procedures; the analyzed documents are listed in Table 1. As mentioned, processes in penitentiary mental health division are not formally documented; two internal documents (i.e., prisoner entry sheet and protocol for the reduction of self-harm and suicidal risk of prisoners) were analysed; all the other analysed documents concern national regulations of penitentiary mental health service and national and regional provisions to contrast and contain the Covid-19 epidemiological emergency. Finally, one of the authors, as Coordinator of the Penitentiary Mental Health Service, participated to all the instances of the process carried out in the period 1–30 May 2020 to understand how activities were performed.

**Table 1:** List of analyzed documents.

Document	Issue Date	Description	Organizations involved
Decree of the President of the Council of Ministers (DPCM)	April 1st, 2008	Procedures and criteria for the transfer of resources, employment relationships, tools and equipment from Penitentiary Administration to National Health Service	National Health Service Penitentiary Administration Regions
Unified conference	January 22, 2015	Guidelines on healthcare delivery for adults in penitentiary; implementation of regional and national healthcare network	Regions Local Health Authority Penitentiary Administration
Memorandum of understanding Bari Local Health Authority/Bari Penitentiary Institute	January 9, 2013	Protocol for the reduction of self-harm and suicidal risk of prisoners and internees in the Bari Penitentiary Institute.	Bari Local Health Authority Bari Penitentiary Administration
Prisoner entry sheet	-	Form filled in by doctor on call and psychologist of new entrant service	Penitentiary Health area Treatment area
National Action Plan for Mental Health (PANSM)	2013		Italian Mental Health Departments
Apulia Region flow chart	March 21, 2020	Covid-19 Health Emergency ‐ Operating Protocols and Flow chart ‐ Update	Local Health Authorities in Apulia region
Protocol note 123/2020 Director of the Health Area of Bari Penitentiary Institute	April 15, 2020	Covid-19 Risk stratification–Operative indications of Bari Penitentiary Health Area	Healthcare personnel of Bari Penitentiary Institute
Decree of the President of the Council of Ministers (DPCM)	April 26, 2020	Further implementing provisions of the Law Decree of 23 February 2020, n. 6, containing urgent measures regarding the containment and management of the epidemiological emergency from Covid-19 applicable throughout the country	Regions
Ministry of Health guidelines	April 23, 2020	Emergency indications for care activities and prevention and control measures in the Mental Health Departments and the Infantile Neuropsychiatry Services	Regions
Regional provisions	April 30, 2020	Provisions on hospitalization activities, measures for the prevention, contrast and containment of the Covid-19 epidemiological emergency; provisions on healthcare delivery and access to healthcare facilities in Apulia Health Service	Local Health Authorities in Apulia region
Note of Bari Local Health Authority–Mental Health Department	May 4, 2020	Resumption of care activities–operative indications	Mental Health Department

**Fig 1:**
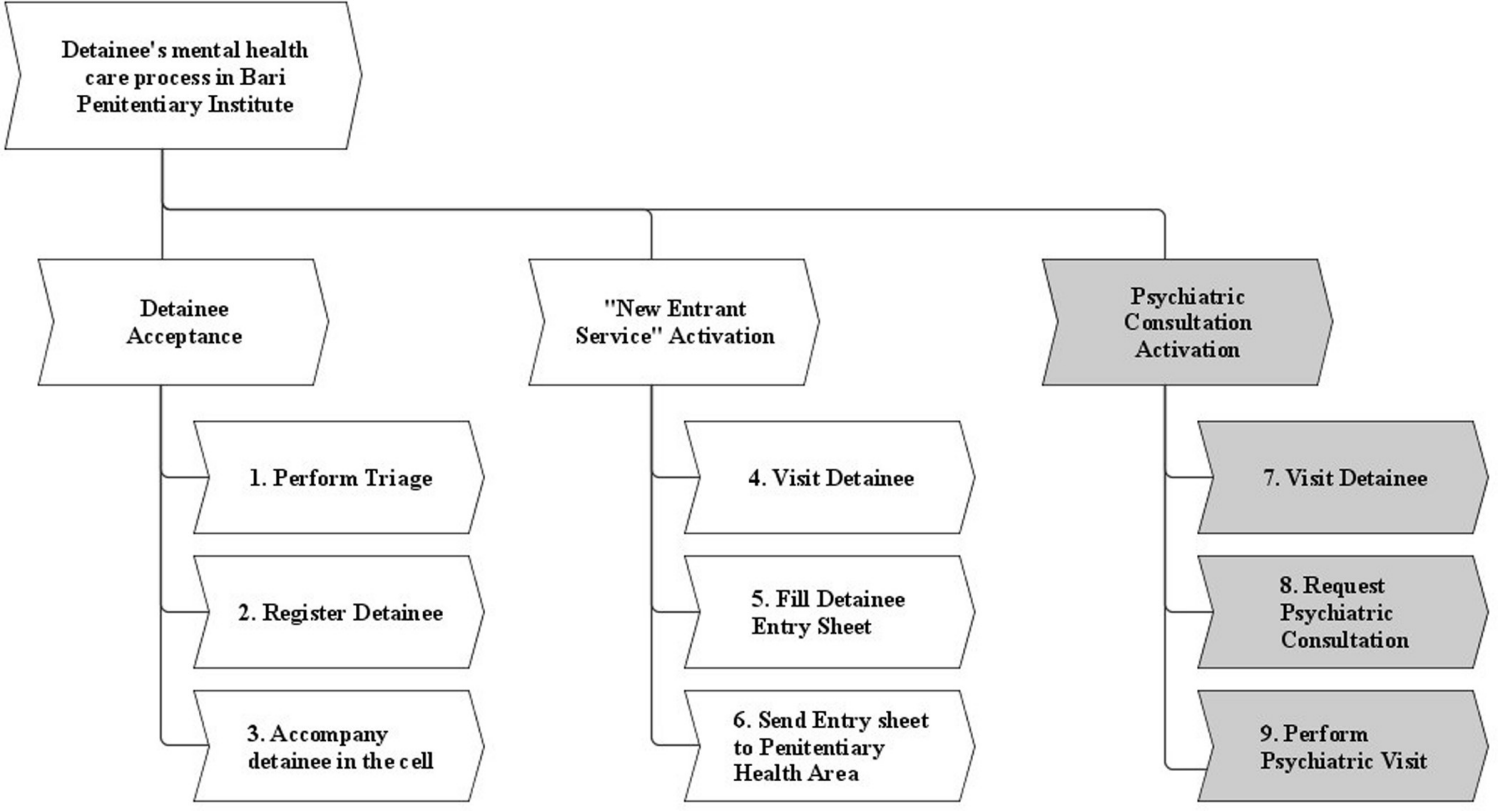
Detainees’ mental health care process Work Breakdown Structure.

To depict the activities performed when a new detainee arrives in the Bari Penitentiary institute and the mental health care process is activated, a Work Breakdown Structure (WBS) was developed. The WBS is a graphical tool that can be used to facilitate (and graphically represent) the top-down decomposition of a project or process into more elementary elements or components. As shown in Fig 1, the first macro activity concerns the detainee acceptance: when a new detainee enters Bari Penitentiary Institute, he/she is registered (activity 2) and then accompanied to the cell (activity 3). This macro activity does not involve personnel of penitentiary mental health division and it has been aligned to the Covid-19 prevention indications by introducing a preliminary step of Covid-19 triage for all new detainees (activity 1), the isolation of new entrants in a section of the penitentiary institute as well as the introduction of personal protective equipment for penitentiary staff. The second macro activity is the “new entrant service”: for each new detainee, within 24 hours, the psychological conditions are assessed by a psychologist of “new entrant service” who visits the detainee (activity 4), fills in a detainee’s entry sheet (activity 5) and send it to the doctor of penitentiary health area (activity 6) who is responsible of detainees’ global health. The “new entrant service” is performed by a psychologist that belongs to penitentiary mental health division; this macro activity does not present criticalities as it has already been adapted to Covid-19 prevention indications by introducing personal protective equipment for penitentiary staff and social distance measurements. The third macro activity concerns the activation of psychiatric consultation: the doctor of penitentiary health area visits the detainee (activity 7) and, taking into the account the psychologist’s evaluations, requests, if needed, a psychiatric consultation (activity 8); the detainee is then visited by the psychiatrist of mental health division. Furthermore, the psychiatric service consultation is a critical activity since it may be triggered at any time throughout the detention when a detainee’s psychiatric problem or mental disorder is observed. The interviews with the actors of the process and the direct observation of several instances of the process revealed that such macro activity presents many criticalities both related to Covid-19 infection risk and to the existing organizational conventions and practices in the penitentiary institute. For these reasons, the detainees’ mental health care process was restricted to the activities concerning the psychiatric consultation, so limiting the process analysis to the activities that start from the identification of a detainee’s psychiatric need and end when detainee is taken in care.

Once the boundaries of the analysis were defined, the root definition [107] was derived as follows:

*The detainees’ mental health care process is carried out to address a need of the prisoners; the ownership of the process is Bari Local Health Authority*, *and the process involves actors belonging to different penitentiary departments (i*.*e*., *Penitentiary Administration*, *Penitentiary Health Area and Penitentiary Mental Health division)*. *The care process is generally triggered by the doctor of penitentiary health area and satisfies the detainee’s psychiatric healthcare need*. *The process starts when a psychiatric healthcare need is identified; process’s output is the psychiatric healthcare need satisfied (thus the detainees taken in care by the penitentiary mental health care service)*. *The objective of process redesign istwofoldd*: *(i) aligning the management of the process to Covid-19 prevention national and regional provisions and protocols and (ii) making the process compliant with national regulations introduced in 2008 that transferred healthcare expertise from penitentiary administration to National Healthcare Service*.

A succinct description of the problem situation to be addressed, process boundaries and motivation of the analysis (CATWOE) is reported in Table 2.

**Table 2:** Root definition for the detainees’ mental health care process.

**C**ustomers	Detainees
**A**ctors	Psychiatry, penitentiary police commander, penitentiary police officer, doctor of penitentiary health area
**T**ransformation	Detainee’s (suspected) psychiatric healthcare need identified → psychiatric healthcare need satisfied (detainee taken in care)
**W**orldview	• need to make the process compliant with penitentiary national and regional regulation • need to safely provide the psychiatric healthcare service to detainees affected by mental disorders in the Covid-19 pandemic situation
**O**wner	Bari Local Health Authority
**E**nvironment	Institutions and departments involved; existing organizational practices and conventions; Covid-19 emergency

All data and information collected allowed to describe the as-is process; to this end, all the activities included in the detainees’ mental health care process were characterized (Table 3).

**Table 3:** Process activities.

Activity ID	Activity	Input	Output	Actors	Resources
7.1	Visit detainee	Detainee from new entrant service arrived	Psychiatric healthcare need identified/not identified	Doctor of penitentiary health area	Personal protective equipment
7.2	Decide how to proceed	Psychiatric healthcare need identified	Decision to communicate to penitentiary police commander/decision to request psychiatric consultation	Doctor of penitentiary health area	
8.3	Communicate suspected psychiatric healthcare need to police commander	Suspected psychiatric healthcare need identified	Suspected psychiatric healthcare need communicated	Penitentiary Police Officer	Telephone
8.1	Request psychiatric consultation and fill consultation register	Decision to request psychiatric consultation	Psychiatric consultation requested	Doctor of penitentiary health area	Consultation register
8.2	Communicate psychiatric healthcare need to police commander	Decision to communicate to penitentiary police commander	Psychiatric healthcare need communicated	Doctor of penitentiary health area	Telephone
8.4	Request psychiatric consultation and call psychiatrist	Suspected psychiatric healthcare need communicated/Psychiatric healthcare need communicated	Psychiatric consultation requested	Penitentiary Police Commander	Telephone
9.1	Plan psychiatric visit	Psychiatric consultation requested	Psychiatric visit planned	Psychiatrist	Consultation register
9.2	Communicate the time of the visit to police commander	Psychiatric visit planned	Time of the visit communicated	Psychiatrist	Telephone
9.3	Arrange detainee’s transfer	Time of the visit communicated	Transfer arranged	Penitentiary police commander	
9.4	Take detainee to visit	Transfer arranged	Detainee transferred	Penitentiary police officer	
9.5	Look for interview space	Time of the visit arrived	Interview space identified	Psychiatrist	Interview space
9.6	Wait until the interview space is free	Interview space identified	Interview space free	Psychiatrist	Interview space
9.7	Occupy interview space	Interview space free	Interview space occupied	Psychiatrist	
9.8	Perform psychiatric visit	Detainee arrived; Interview space occupied	Psychiatric healthcare need confirmed and detainee taking in care/Psychiatric healthcare need not confirmed	Psychiatrist	Interview space

**Fig 2:**
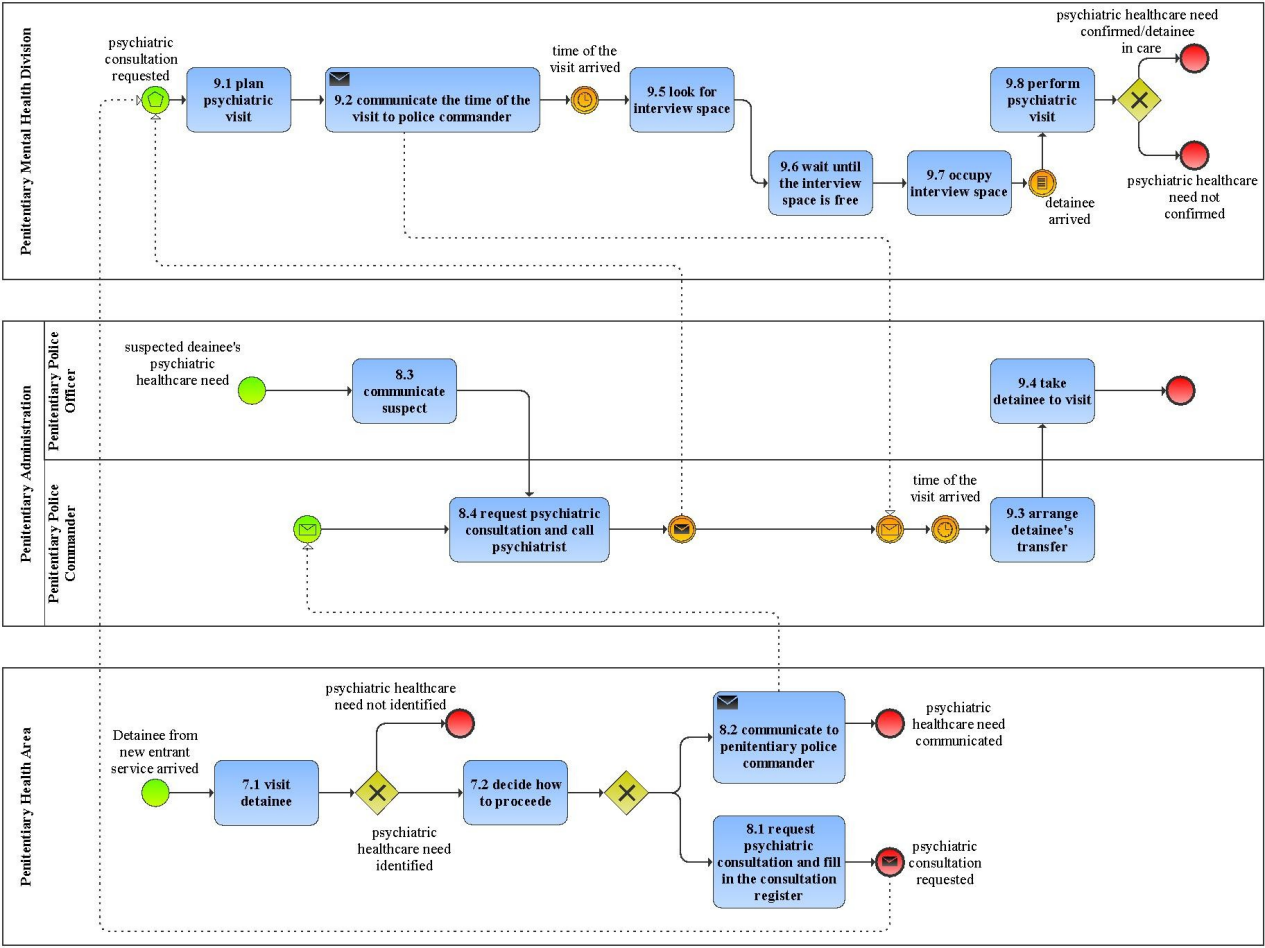
As-is process model of detainees’ mental health care process.

The process of detainees’ mental health care was modelled in BPMN (Fig 2). The as-is process model was validated by all actors involved in the process. The model was also discussed with the actors to identify inefficiencies, problems and criticalities of the process.

The analysis of as-is model revealed that some activities were characterized by a high Covid-19 infection risk because not aligned with national and regional provisions for the prevention and containment of Covid-19 infection. After a period of completely limitation of all health services only to the emergency ones, including no-emergency psychiatric consultation in penitentiary context, the Italian Ministry of Health issued guidelines for the reactivation of all these healthcare activities in particular contexts as mental health departments. The issued guidelines, adopted by Apulia Region and Bari Local Health Authority, introduced indications for the prevention of Covid-19 infection risk in Mental Health Department. Staff and healthcare personnel was indeed particularly exposed to Covid-19 infection risk due to the direct, close and prolonged contact with patients. Ministry of Health indications that should be respected in penitentiary mental health division to prevent Covid-19 infection risk are reported in Table 4 with a comment about the as-is situation in Bari penitentiary mental health division.

**Table 4:** Ministry of Health indications and adoption state in Bari penitentiary mental health division.

Indication	Indication adoption in AS-IS situation	AS-IS criticalities
All patients should undergo Covid-19 triage before psychiatric visit	Indication respected	-
Safety distance must be greater than 2 m and sufficient aeration should be guaranteed during the visit	Indication not respected	Interview spaces are cells (of about 4m^2^) that do not guarantee an adequate aeration and social safety distance
Number of contacts during healthcare activities has to be minimized	Indication not respected	Number of contacts during healthcare activities is always included between 6 and 12; contacts occur especially when psychiatrist looks for interview space and waits for the release of interview space (that are shared with other activities)
Patient should wear a mask	Indication not respected	When the detainee is visited by psychiatrist, he/she does not wear any mask
Psychiatrist has to wear the max level of personal protection equipment in case of suspected Covid-19 case	Indication not respected	Psychiatrist does not wear an adequate level of personal protection equipment when visit detainee because he/she does not know Covid-19 level of risk of each detainee that he/she visits

The comparison of as-is situation of the detainees’ mental health care process with the provisions provided by Ministry of Health for the prevention of Covid-19 infection risk in mental health department, together with the direct observation and discussion with the actors of the process, allowed to detect the activities with the greatest level of Covid-19 risk: “perform psychiatric visits”, “look for interview space” and “wait until the interview space is free”. The Covid-19 infection risk in the Penitentiary is due to organizational problems and inefficiencies; for instance, the “perform psychiatric visits” activity presents inefficiencies because detainee and psychiatrist do not wear adequate protection as well as the interview spaces are not adequate to guarantee safety social distance; additionally, the high level of Covid-19 risk is due to the communication of Covid-19 level of risk for each detainee that is not effectively performed. The activities “look for interview space” and “wait until the interview space is free” are also risky activities as the visit spaces are shared: that causes long waits for the psychiatrist and increases the probability of contact with other people during the execution of the activities.

Another important criticality concerns the request of psychiatric consultation. In the as-is process this activity is very confusing as the actors that should perform it are not clearly defined. As shown in Fig 2, the doctor of the penitentiary health area, in some cases, rather than directly asking for the psychiatric consultation, communicates the need of psychiatric consultation to the police commander who then demands the psychiatric consultation; this activity does not add any value to the process; moreover, it is not managed accordingly to current regulation that assigns the responsibility of detainees’ health exclusively to penitentiary health area. Also, in the as-is process, when a penitentiary police officer identifies a detainee that could be affected by mental disorder, communicates the suspected detainee’s psychiatric healthcare need to the penitentiary police commander who directly requires the psychiatric consultation, without involving the doctor of penitentiary health area. This activity is carried out based on old organizational practices and conventions (the penitentiary regulation in force before 2008 attributed health care expertise to penitentiary administration).

### Detainees’ mental health care to-be process

The identified critical issues and inefficiencies, both related to Covid-19 risk and related to existing organizational conventions and practices, led to the redesign of the detainees’ mental health care process with the aim to reduce Covid-19 risk and make it more compliant to current national penitentiary regulation.

After some meetings with process actors and the Director of penitentiary health area and the Director of Bari Penitentiary institute, feasible and desirable changes to improve the process and reduce Covid-19 risk were identified. Table 5 shows the proposal formulated to overcome the main criticalities and inefficiencies of detainees’ mental health care process.

**Table 5:** As-is process criticalities and to-be proposal.

Activity	AS-IS criticalities	TO-BE proposal
Request psychiatric consultation	Lack of a standard protocol; not compliance with the current penitentiary regulation which attributes the responsibility of detainees’ health exclusively to penitentiary health area	Definition of standard protocol to carry out the activity
Perform psychiatric visit	Interview spaces are cells (of about 4m^2^) that do not guarantee adequate aeration and social safety distance	Identification of a more suitable and larger interview spaces
	When the detainee is visited by psychiatry, he/she does not wear any mask	Provision of masks to detainees
	Psychiatrist does not wear an adequate level of personal protection equipment during the visit because he/she does not know Covid-19 level of risk of each detainee	Assessment of Covid-19 level of risk for each detainee Provision of adequate personal protection equipment to psychiatrists
Look for interview space Wait until the interview space is free	Number of contacts during healthcare activities is always in between 6 and 12; contacts occur especially when psychiatrist looks for interview space and waits for the availability of interview space (that are shared with other activities)	Provision of adequate personal protection equipment to psychiatrists Identification of interview spaces exclusively dedicated to psychiatric activities Introduction of technological device to improve communication among operators of different divisions (e.g., communication between psychiatrist and penitentiary police officer/commander)

To overcome the criticalities related to the request of psychiatric consultation (that, as mentioned, is not compliant with the current penitentiary regulation) the need of a coordination mechanism to better manage interdependence between the process activities arose. A standardization coordination mechanism, the less costly coordination mechanisms [110], was suggested. The activities “look for interview space”, “wait util interview space is free” and “perform psychiatric visit” are indeed risky activities because the resource they use (the interview space) is shared with other activities of other processes performed in the prison. To overcome the criticalities of these activities characterized by great level of Covid-19 risk, other redesign strategies were considered. For instance, the introduction of new value-added activities modifying the process workflow, the assignment of new activities and responsibilities to process actors as well as the introduction of digital technologies. During some meetings with the process actors, the following proposals were presented:

- Definition of standard protocol to carry out the request of psychiatric consultation;
- Identification of an interview space exclusively dedicated to psychiatric visit. The space had to be enough large (more than 8 m^2^) to guarantee correct aeration and social safety distance during the psychiatric visit;
- Assessment, by the doctor of penitentiary health area, of Covid-19 level of risk for each detainee and transcription in the consultation register;
- Provision of masks to detainee and adequate personal protection equipment to psychiatrists;
- Introduction of technological devices, e.g. a pager, to improve communication among operators working in different divisions (e.g., communication between psychiatrist and penitentiary police officer/commander) so as to reduce direct contacts.

**Fig 3:**
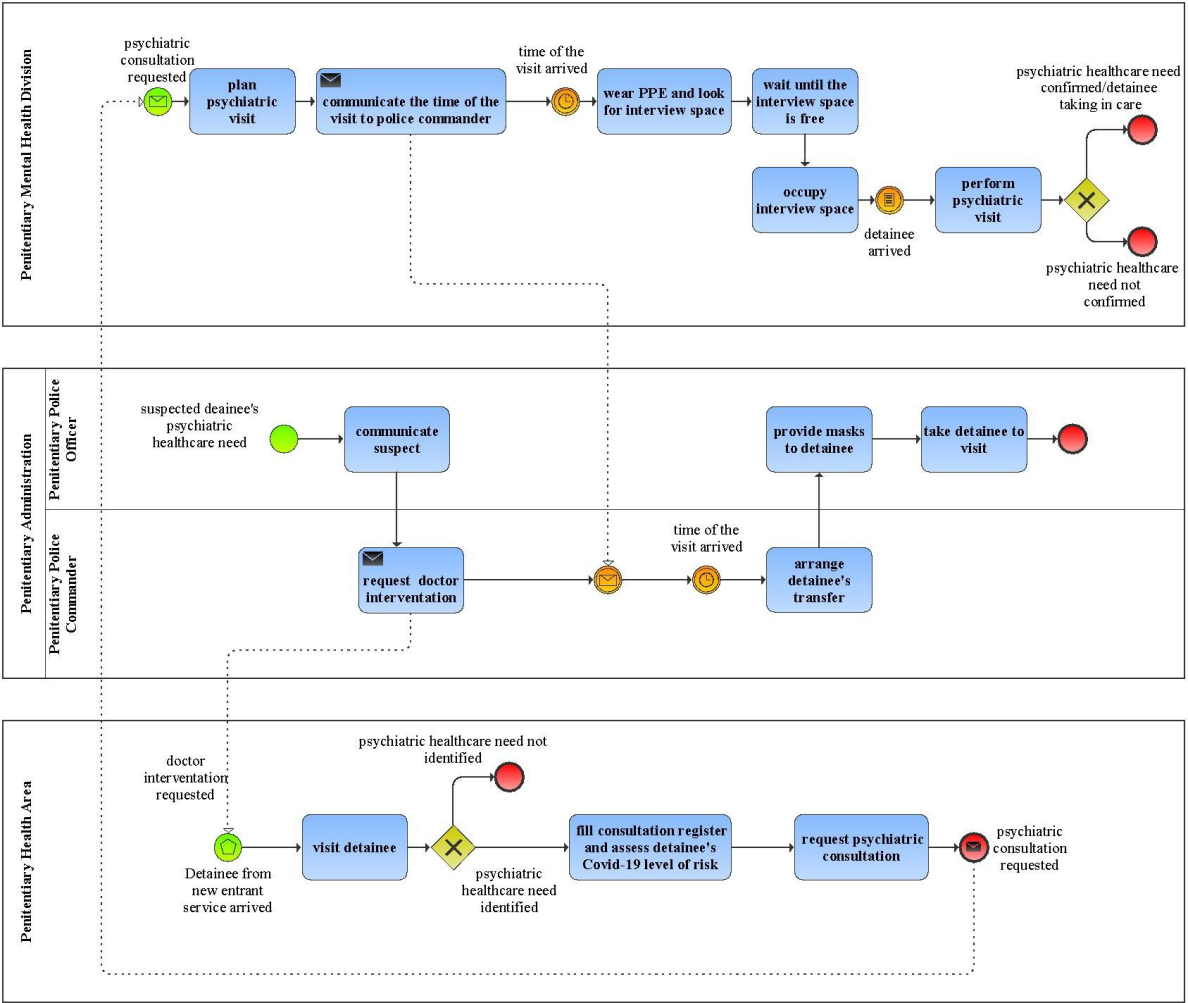
To-be process model of detainees’ mental health care process.

A standard operative protocol was defined and enacted to manage the request of the psychiatric consultation so as to make it compliant with current national penitentiary regulation. To provide adequate personal protection equipment to psychiatrist was considered a feasible change to improve the Covid-19 risk of the activities “looks for interview space” and “wait until the interview space is free”. The proposal to identify an interview space completely dedicated to psychiatric activities was considered unfeasible because of the limited availability of spaces in the prison. Similarly, the proposal to introduce technological devices (e.g., a pager) to improve communication among operators belonging to different divisions (e.g., communication between psychiatrist and penitentiary police officer/commander) and reduce direct contacts was considered unfeasible for security and safety reasons. Based on that, a single to-be alternative was identified and implemented. The to-be process was also modelled in BPMN (Fig 3). In the redesigned process the workflow was modified by adding activities to reduce Covid-19 infection risk. For instance, the activity “fill consultation register and assess detainee’s Covid-19 level of risk” was added. The responsibility was assigned to the doctor of Penitentiary Health Area. Additionally, as mentioned, a standard operative protocol to manage the request of psychiatric consultation was introduced. It clearly defined roles and responsibilities of process’s actors. Based on that, some no value-added activities, e.g. the possibility for the staff of Penitentiary Administration to directly require psychiatric consultation without involving healthcare personnel and “communicate to penitentiary police commander” performed by the doctor of penitentiary health area, were eliminated to make the process straightforward and compliant with national penitentiary regulation. The responsibility to request the psychiatric consultation was assigned exclusively to the doctor of penitentiary health area, as envisaged by current national penitentiary regulation.

The to-be process was implemented and tested on 13 process instances.

To evaluate the Covid-19 risk reduction associated to the to-be process and compare it with the as-is process, an indicator related to Covid-19 infection risk in the penitentiary context was developed. The average Covid-19 infection risk (R_a_) for each process activity *a* was defined as follows:

$$R_{a}=1-\prod_{i=1}^{n}\left(1-R_{i}\right)$$

where:

- *n* is the number of risky contacts, namely the number of subjects that the psychiatrist meets when performs an activity
- *R*_*i*_ is the Covid-19 infection risk associated to the i-th contact. It depends on the following Covid-19 risk factors: social distance (R_dist_), contact time (R_t_) and presence of personal protection equipment (R_ppe_). *R*_*i*_ is calculated as follows:

$${\text{R}}_{\text{i}}={\text{R}}_{\text{ppe}}*{\text{R}}_{\text{dist}}*{\text{R}}_{\text{t}}$$

Based on extant literature and Ministry guidelines, different conditions were considered to reasonably estimate Covid-19 risk factors, as shown in Table 6.

**Table 6:** Covid-19 factor risk estimation.

Risk factor	Conditions	Risk estimation
R_ppe_	FFP3 mask	0.05
	FFP2 mask	0.50
	Other filtering mask	0.60
	Surgical mask	0.80
	No mask	1
R_dist_	dist > 2 m	0.10
	1m ≤ dist ≤ 2m	0.50
	0m ≤ dist ≤1m	1
R_t_	t ≤ 15 min	0.20
	t > 15 min	1

The average Covid-19 infection risk was calculated for the average as-is process instance and all the tested to-be process instances. The changes introduced in the to-be alternative significantly reduce the Covid-19 infection risk for the actors involved in the process, since the risk associated with the activities characterized by a higher level of Covid-19 risk is drastically reduced. For all the tested instances, *R*_*a*_ of such activities is always smaller than the one associated to the attendant activities of the average as-is process instance. Moreover, as mentioned, the to-be process model is compliant with the current national penitentiary regulation.

## Results and discussion

After the successful testing stage on the 13 pilot instances, the to-be process became the new as-is. The Penitentiary Director issued a service order to regulate the provision of personal protection equipment, to regulate the dimensions of the interview spaces so as to guarantee proper aeration and social distancing, to regulate the communication of detainees’ Covid-19 level of risk. The to-be process led to an important reduction of Covid-19 infection risk for all the activities performed. Also, as mentioned, a standard operative protocol was discussed, approved, and implemented to manage the “request psychiatric consultation” so as to be compliant with current regulation.

The process criticalities were identified and addressed by adopting Soft System Methodology together with Business Process Management approach. To the best of the authors’ knowledge, none of the two approaches has been applied to improve processes in the penitentiary context. Moreover, the combined use is quite innovative: it allowed a human-centered process-based redesign approach to be adopted while overcoming the limits of each approach.

The process-based approach to analyse and redesign a problem situation is in line with the process theory according to which reality can be represented as a chain of activities and events that are activated to deliver a specific outcome. BPM enables the process-based (re)design approach by providing methods and techniques for representing reality as a chain of events and activities (also defined processes) and detecting criticalities that affect it (in terms of inefficient process workflow, not effective coordination mechanisms between process’ activities, and not adequate information and documentary flows).

The adoption of a human-centered approach to progress is one of the key concepts of Society 5.0, the vision on the future of society that inspired the new emerging paradigm of Industry 5.0 recently proposed by the European Commission [111, 112]. Under such an approach, any transformation should be designed and managed according to human needs rather than being based on purely technical and economic perspectives [113]. The participatory approach adopted goes in such a direction. The SSM learning cycle indeed stimulates the active participation of all the actors involved and affected by a problem situation. The actors are invited to identify solutions that are desirable and feasible [107]. However, SSM does not envisage any specific technique to build conceptual models based on the root definition, compare models with the real world and foster discussion to make desirable and feasible solutions to arise. BPM provides methods and techniques to support such aspects. For example, Process modelling techniques (e.g., BPMN) as well as the redesign strategies suggested to improve a process can be used to overcome that limit. Also, the focus on testing and monitoring suggested by BPM is useful to successfully implement the step 7 of SSM.

Although successfully adopted in the specific case, the use of the combined approach was also challenging. The different background of the actors involved in the process (e.g., healthcare personnel, director of penitentiary institute and penitentiary police) and the members of the project team devoted to facilitating the process analysis and redesign makes the exchange of information and communications not easy. To make the actors involved in the process appreciate the approach and understand it, training sessions had to be managed. Moreover, the implementation of desirable solutions was not easy. Many of them were considered not feasible. This may occur in the penitentiary context wherein organizational practices and conventions are often established and any change is hard to be accepted. The implementation of the to-be process on pilot instances, despite the limited changes to the process workflow, was not easy. Some actors tended to perform their activities as they always did; for instance, the penitentiary police commander continued to directly require psychiatric consultation for disturbing detainee without involving penitentiary health area. However, after the testing stage (13 pilot instances), the reluctance was addressed, and the to-be process became the new as-is.

## Conclusions

In the study an innovative approach that combines Soft System Methodology and Business Process Management is applied to analyse and redesign the detainees’ mental health care process in Bari Penitentiary Mental Health Division. The process is particularly relevant in the Covid-19 pandemic scenario as it guarantees continuity of psychiatric assistance to prisoners and permit the detection of psychiatric disorders that are considered vulnerability factor for Covid-19 severe illness. The direct, close and prolonged contact with patients exposes detainees and psychiatric staff to a high Covid-19 risk and makes the process very critical.

The analysis showed that the process is characterized by high Covid-19 infection risk due to organizational inefficiencies associated to: (i) not adequate structural resources to maintain safety social distance (ii) not adequate personal protective equipment for detainees and psychiatrists (iv) waits that expose the psychiatrists to risky contacts. Moreover, the process lacks a standard procedure (responsibilities are not clear) to request psychiatric consultation. Hence, the process was redesigned to align it to Covid-19 prevention regulations and reduce Covid-19 infection risk. The to-be process is also compliant with national regulations introduced in 2008 that transferred healthcare expertise from Penitentiary Administration to National Healthcare Service and was not properly implemented so far.

From a theoretical perspective, the methodological approach proposed–which innovatively combines BPM and SSM–can be used to analyse and redesign other processes both in penitentiary context and in other contexts. The methodological approach allows to overcome the limitations of the application of SSM in healthcare context, wherein the SSM seems to be quite useful to define and structure the problem situation and explore the potential solutions while less useful to put these improvement suggestions in place [78]. On the other hand, BPM methods and techniques (e.g., the redesign strategies as well as the test and monitoring of suggested solution on pilot process instances) are useful to support the implementation of improvements and changes. The advantages derived from the combination of SSM and BPM make the methodological approach suitable for the analysis and redesign of those processes where the human needs are predominant and fundamental. Moreover the study contributes to shed some light on the penitentiary healthcare systems, a highly complex and often neglected domain.

From a managerial perspective, the improvement solutions and organizational changes proposed could be adopted to redesign and improve other similar processes in other penitentiary organizations.

As to the limitations, the innovative methodological approach is implemented on a single case and in a particular context, namely the penitentiary domain. Future research will include the application of the methodological approach to analyse and improve processes in other healthcare settings as well as other organizational contexts.
